# Management and Outcomes in Patients with Breast Cancer with 1-of-1 and 2-of-2 Positive Sentinel Nodes

**DOI:** 10.1245/s10434-025-18097-9

**Published:** 2025-08-23

**Authors:** Walker Lyons, E. McAuley Fish, Richard J. Bleicher, Cecilia Chang, Allison A. Aggon, Alycia L. So, Andrea S. Porpiglia, Austin D. Williams

**Affiliations:** 1 Department of Surgical Oncology, Fox Chase Cancer Center, Philadelphia, PA USA; 2 NorthShore University Health System Research Institute, Evanston, IL USA

## Abstract

**Background:**

The ACOSOG-Z0011 and AMAROS trials showed that axillary lymph node dissection (ALND) provided no benefit for patients with 1–2 positive sentinel lymph nodes (+SLNs). There remains apprehension to omit ALND for patients in whom only 1–2 SLNs are retrieved and all are positive. This study evaluates current practices and pathological findings when ALND is pursued.

**Patients and Methods:**

We identified female patients with cT1–3N0 breast cancer who underwent sentinel lymphadenectomy from 2018 to 2021 in the National Cancer Database (NCDB). Patients with ≤ 2 SLNs were included and categorized on the basis of the number positive/removed: 0/1, 1/1, 1/2, and 2/2. We assessed the rates and factors associated with ALND.

**Results:**

A total of 102,802 patients were included: 0/1: 79,106 (77%), 1/1: 10,549 (10%), 1/2: 10,068 (10%), and 2/2: 3079 (3%). ALND was most frequently performed for patients with 2/2 +SLNs (41%), followed by 26% with 1/1 +SLNs, 19% with 1/2 +SLNs, and 6% with 0/1 +SLNs. On multivariable analysis, 2/2 +SLN status was the strongest independent predictor of ALND. For triple-negative and human epidermal growth factor receptor (HER)2+ patients, ALND did not affect adjuvant chemotherapy or radiation rates. Among pN+ hormone receptor (HR)+/HER2− patients > 50, ALND was linked to higher chemotherapy rates in all SLN groups, despite no difference in 21-gene recurrence scores. With a median follow-up of 35.4 months, ALND did not improve overall survival.

**Conclusions:**

ALND is being performed at higher-than-expected rates in patients with ≤ 2 +SLNs and may contribute to adjuvant overtreatment, particularly in HR+/HER2− patients. Multidisciplinary case discussions and ongoing provider education are essential to reduce unnecessary axillary interventions.

**Supplementary Information:**

The online version contains supplementary material available at 10.1245/s10434-025-18097-9.

Axillary lymph node management in breast cancer treatment has changed significantly over the past 30 years. While ALND was felt to help achieve local control, it is also associated with increased morbidity, specifically high rates of lymphedema, paresthesia, seroma formation, and infection, along with decreased shoulder mobility. Beginning with the introduction of sentinel lymphadenectomy (SLNB) in the 1990s, the shift away from axillary lymph node dissection (ALND) began.^1–4^ After the initial adoption of SLNB, ALND was still being performed whenever a positive node was found until the landmark American College of Surgeons Oncology Group Z0011 (Z0011) trial challenged this practice. Z0011 found that ALND could be safely omitted in clinically node-negative patients undergoing upfront breast conservation (BCT) when only one or two positive sentinel lymph nodes (+SLNs) were found.^5^ With the publication of the European After Mapping of the Axilla: Radiotherapy Or Surgery (AMAROS) trial, which included 17% of patients who had mastectomy, this paradigm began extending into non-BCT cases.^6^ Since then, other large, randomized controlled studies such as the SINODAR-ONE and SENOMAC trials have validated these results and expanded them to include patients with clinical T3 tumors, and those with extracapsular extension (ECE).^7,8^

With these practice changing trials, rates of ALND have decreased.^9,10^ Yet surgeons (and others on the care team) remain hesitant to omit ALND in several clinical situations. One such circumstance is when fewer than three SLNs are retrieved, and all are positive out of fear of missing additional axillary disease. It is possible that this hesitation is influenced by the unease of knowing whether a third SLN, if retrieved, might be positive, although the mean number of SLNs removed in Z0011 and AMAROS was two. Neither of these trials, however, report the proportion of enrolled patients who had metastatic disease present in all retrieved SLNs.

To date, there are no studies that have assessed current practice patterns when only one or two SLNs are retrieved and all are positive, nor are there data to inform clinicians about the patients most likely to harbor clinically relevant axillary disease when completion ALND (cALND) is performed. This study aims to fill this gap in the literature and hypothesized that cALND is being performed commonly for these patients, that rates of additional axillary disease are similar to those found in randomized trials, and that performance of ALND does not have an impact on survival.

## Patients and Methods

### Patient Cohort and Study Variables

We performed a retrospective analysis of the National Cancer Database (NCDB), a joint effort of the American College of Surgeons and the American Cancer Society. The NCDB contains deidentified patient-level data from more than 1500 Commission-on-Cancer accredited programs and captures approximately 70% of all malignancies in the United States.^11,12^

From the NCDB breast cancer Participant User File, we identified female patients ≥ 18 years of age who underwent upfront surgery with SLNB for their first clinical tumor (cT)1–T3, N0, M0 breast cancer between 2018 and 2021 (Supplementary Fig. 1). Patients treated prior to 2018 were not included in the analyses because 2018 is the first year that the NCDB began recording specific SLN information such as the number of nodes removed and the number positive for metastasis. We excluded patients if they had clinically occult breast cancer, if their breast or axillary surgery was unknown, and if they had any form of neoadjuvant therapy. Finally, we excluded patients who had > 2 SLNs removed, and those with 2 SLNs retrieved with all being negative.

Patients were then grouped on the basis of the number of SLNs positive/removed: 0/1, 1/1, 1/2, and 2/2. We compared demographic and clinicopathological characteristics and surgical approach between SLN groups. The proportion of patients who underwent ALND was calculated, and among these patients the total number of lymph nodes (LNs) examined and positive were compared. The use of ALND over time was analyzed for all SLN groups. We assessed the factors within the overall cohort associated with receipt of ALND, and among those undergoing ALND, the factors associated with finding additional +LNs.

Subgroup analyses of surgical approach and adjuvant therapies were performed on the basis of receptor subtype. For patients with hormone receptor (HR) positive, human epidermal growth factor receptor 2 (HER2) negative breast cancer, we also analyzed the use and results of 21-gene recurrence score testing (Oncotype DX, Genomic Health) in the overall group and in groups stratified by age (≤ 50 and > 50) as a surrogate for menopausal status (as was done in the RxPONDER study when menopausal status was unknown).^13^

Finally, we performed analyses of overall survival (OS). Unadjusted OS was compared among SLN group according to ALND receipt.

### Statistical Analysis

Comparisons between groups were made using chi-squared test, Student’s *t*-test, and analysis of variance (ANOVA), as appropriate. Univariate and multivariable logistic regression models, adjusting for pertinent clinicopathologic and demographic features, were performed. The Kaplan–Meier method and log-rank tests were used to analyze OS. Statistical tests were two-sided, and a *p*-value < 0.05 was considered significant. All analyses were performed using SAS version 9.4 (SAS Institute, Inc., Cary, NC, USA). The study was deemed exempt by the Fox Chase Cancer Center Institutional Review Board.

## Results

### Demographics and Clinicopathologic Findings

In total, 179,918 patients with cT1-3N0 breast cancer who underwent upfront surgery with SLNB and had < 2 SLNs removed were identified (Supplementary Fig. 1). After excluding 77,116 patients with 0/2 +SLNs, we were left with an analysis cohort of 102,802 patients. Patients were stratified by the number of +SLNs and total number of SLNs removed. Most patients (*n* = 79,106, 77%) had one negative LN removed (0/1) while 10% (*n* = 10,549) had 1/1 + SLNs, 10% (*n* = 10,068) had 1/2 +SLNs, and 3% (*n* = 3079) had 2/2 +SLNs (Table 1). Patients in the 0/1 +SLNs group were older and more likely to have cT1 tumors, ductal histology, and low-grade and absent lymphovascular invasion (all* p* < 0.001) when compared with the other SLN groups. Conversely, patients with 2/2 +SLNs were more likely to have cT3 tumors, lobular histology, high tumor grade, and presence of lymphovascular invasion (all *p* < 0.001). A lower proportion of patients with HR+/HER2− cancers had 0/1 +SLNs when compared with patients with HER2+ and triple negative breast cancers (TNBC) (76% versus 81% and 85%, respectively, *p* < 0.001). There was very little difference between the SLN groups based on race/ethnicity, Charlson–Deyo score, insurance status, and high school education.

**Table 1:** Clinicodemographic features

	Overall	Sentinel lymph node status (positive/removed)				*p*
		0/1	1/1	1/2	2/2	
*N*	102,802	79,106	10,549	10,068	3079	
Age at diagnosis, years [median (IQR)]	63 (52–74)	64 (53–75)	61 (49–73)	60 (48–72)	60 (48–72)	< 0.001
*Race/ethnicity*						< 0.001
NH white	80,219 (78.0)	62,225 (78.7)	8045 (76.3)	7562 (75.1)	2387 (77.5)	
NH Black	8822 (8.6)	6604 (8.4)	1004 (9.5)	942 (9.4)	272 (8.8)	
Hispanic	6077 (5.9)	4476 (5.7)	686 (6.5)	710 (7.1)	205 (6.7)	
Asian	4998 (4.9)	3768 (4.8)	515 (4.9)	572 (5.7)	143 (4.6)	
Other/unknown	2686 (2.6)	2033 (2.6)	299 (2.8)	282 (2.8)	72 (2.3)	
*Charlson–Deyo score*						0.06
0	83,576 (81.3)	64,164 (81.1)	8576 (81.3)	8294 (82.4)	2542 (82.6)	
1	13,232 (12.9)	10,288 (13.0)	1336 (12.7)	1242 (12.3)	366 (11.9)	
≥ 2	5994 (5.8)	4654 (5.9)	637 (6.0)	532 (5.3)	171 (5.6)	
*Insurance status*						< 0.001
Not insured	992 (1.0)	670 (0.9)	133 (1.3)	153 (1.5)	36 (1.2)	
Private insurance	49,445 (48.1)	36,966 (46.7)	5381 (51.0)	5444 (54.1)	1654 (53.7)	
Medicaid	5503 (5.4)	3867 (4.9)	741 (7.0)	682 (6.8)	213 (6.9)	
Medicare	44,881 (43.7)	36,113 (45.7)	4070 (38.6)	3575 (35.5)	1123 (36.5)	
Other government	1165 (1.1)	861 (1.1)	135 (1.3)	134 (1.3)	35 (1.1)	
Unknown	816 (0.8)	629 (0.8)	89 (0.8)	80 (0.8)	18 (0.6)	
*No high school degree*						< 0.001
≥ 15.3%	13,799 (13.4)	10,432 (13.2)	1472 (14.0)	1464 (14.5)	431 (14.0)	
9.1–15.2%	22,571 (22.0)	17,244 (21.8)	2396 (22.7)	2218 (22.0)	713 (23.2)	
5.0–9.0%	26,629 (25.9)	20,612 (26.1)	2691 (25.5)	2537 (25.2)	789 (25.6)	
< 5.0%	23,102 (22.5)	17,986 (22.7)	2241 (21.2)	2229 (22.1)	646 (21.0)	
Unknown	16,701 (16.3)	12,832 (16.2)	1749 (16.6)	1620 (16.1)	500 (16.2)	
*Median income*						0.24
< $46,277	10,862 (10.6)	8268 (10.5)	1161 (11.0)	1086 (10.8)	347 (11.3)	
$46,227–57,856	16,815 (16.4)	12,879 (16.3)	1771 (16.8)	1644 (16.3)	521 (16.9)	
$57,857–74,062	20,678 (20.1)	16,002 (20.2)	2050 (19.4)	2027 (20.1)	599 (19.5)	
≥ $74,063	37,580 (36.6)	28,994 (36.7)	3799 (36.0)	3678 (36.5)	1109 (36.0)	
Unknown	16,867 (16.4)	12,963 (16.4)	1768 (16.8)	1633 (16.2)	503 (16.3)	
*Institution type*						< 0.001
Community cancer center	7880 (7.7)	6073 (7.7)	824 (7.8)	768 (7.6)	215 (7.0)	
Comprehensive community cancer program	44,477 (43.3)	34740 (43.9)	4483 (42.5)	3970 (39.4)	1284 (41.7)	
Academic/research program	27,191 (26.5)	20,745 (26.2)	2756 (26.1)	2856 (28.4)	834 (27.1)	
Integrated network cancer program	20,926 (20.4)	16,192 (20.5)	2071 (19.6)	2040 (20.3)	623 (20.2)	
Unknown	2328 (2.3)	1356 (1.7)	415 (3.9)	434 (4.3)	123 (4.0)	
*Clinical tumor stage*						< 0.001
cT1	82,072 (79.8)	66,788 (84.4)	6872 (65.1)	6712 (66.7)	1700 (55.2)	
cT2	19,223 (18.7)	11,656 (14.7)	3304 (31.3)	3092 (30.7)	1171 (38.0)	
cT3	1507 (1.5)	662 (0.8)	373 (3.5)	264 (2.6)	208 (6.8)	
*Receptor subtype*						< 0.001
HR+/HER2−	90,147 (87.7)	68,502 (86.6)	9640 (91.4)	9204 (91.4)	2801 (91.0)	
HER2+	4332 (4.2)	3507 (4.4)	371 (3.5)	339 (3.4)	115 (3.7)	
TNBC	6203 (6.0)	5287 (6.7)	395 (3.7)	401 (4.0)	120 (3.9)	
Unknown	2120 (2.1)	1810 (2.3)	143 (1.4)	124 (1.2)	43 (1.4)	
*Tumor histology*						< 0.001
Ductal	89,202 (86.8)	69,313 (87.6)	8846 (83.9)	8644 (85.9)	2399 (77.9)	
Lobular	12,830 (12.5)	9136 (11.6)	1653 (15.7)	1374 (13.7)	667 (21.7)	
Mixed/other/unknown	770 (0.8)	657 (0.8)	50 (1.4)	50 (0.5)	13 (0.4)	
*Tumor grade*						< 0.001
Low	28,931 (28.1)	24,505 (31.0)	1997 (18.9)	1927 (19.1)	502 (16.3)	
Intermediate	52,118 (50.7)	38,417 (48.6)	6108 (57.9)	5760 (57.2)	1833 (59.5)	
High	19,747 (19.2)	14,386 (18.2)	2332 (22.1)	2307 (22.9)	722 (23.5)	
Unknown	2006 (2.0)	1798 (2.3)	112 (1.1)	74 (0.7)	22 (0.7)	
*Lymphovascular invasion*						< 0.001
Absent	78,341 (76.2)	65612 (82.9)	1136 (10.8)	5704 (56.7)	1364 (44.3)	
Present	13,832 (13.5)	5401 (6.8)	3752 (35.6)	3284 (32.6)	1395 (45.3)	
Unknown	10,629 (10.4)	8093 (10.2)	5661 (53.7)	1080 (10.7)	320 (10.4)	

Data are expressed as *n* (%) unless otherwise specified.

*NH* non-Hispanic, *HR* hormone receptor, *HER2* human epidermal growth factor receptor 2, *TNBC* triple negative breast cancer

^*^Results suppressed: NCDB does not permit aggregate results for cell sizes < 10

### Surgical Approach

Overall, partial mastectomy was more commonly performed than mastectomy (75% versus 25%, Table 2), but mastectomy was more common in the 2/2 +SLN group, when compared with the other SLN groups (48% versus 0/1: 21%, 1/1: 37%, 1/2: 36%, *p* < 0.001). Similar to cT stage, pathologic tumor (pT) stage increased as the number of positive nodes increased (*p* < 0.001). Most patients had both cT1 (80%) and pT1 (73%) tumors. There was, however, a notable upstage rate in the 2/2 +SLN group, from 7% cT3 to 13% pT3 when compared with the other groups (0/1: 1–1%, 1/1: 4–6%, 1/2: 3–5%).

**Table 2:** Surgical approach and pathologic stage

	Overall	Sentinel lymph node status				*p*
		0/1	1/1	1/2	2/2	
*N*	102,802	79,106	10,549	10,068	3079	
*Breast surgery*						
Partial mastectomy	77,529 (75.4)	62,806 (79.4)	6660 (63.1)	6456 (64.1)	1607 (52.2)	< 0.001
Mastectomy	25,273 (24.6)	16,300 (20.6)	3889 (36.9)	3612 (35.9)	1472 (47.8)	
*Pathologic tumor stage*						< 0.001
pT0	84 (0.1)	83 (0.10)	*	*	*	
pTis	126 (0.1)	124 (0.16)	*	*	*	
pT1	75,196 (73.2)	63,020 (79.7)	5472 (51.9)	5463 (54.3)	1241 (40.3)	
pT2	24,273 (23.6)	14,433 (18.3)	4330 (41.1)	4082 (40.5)	1428 (46.4)	
pT3	2436 (2.4)	896 (1.1)	684 (6.5)	468 (4.7)	388 (12.6)	
pT4	123 (0.1)	37 (0.05)	38 (0.4)	31 (0.3)	17 (0.6)	
pTX	188 (0.18)	175 (0.22)	*	*	*	
Unknown	376 (0.37)	338 (0.43)	17 (0.16)	16 (0.16)	*	
*Axillary surgery*						< 0.001
SLNB alone	91,851 (89.4)	74,035 (93.6)	7814 (74.1)	8190 (81.4)	1812 (58.9)	
SLNB then ALND	10,951 (10.7)	5071 (6.4)	2735 (25.9)	1878 (18.7)	1267 (41.2)	
*Results of ALND*						
Additional LNs removed	6.6 ± 6.8	2.8 ± 3.5	10.0 ± 7.2	8.5 ± 7.1	11.2 ± 7.4	< 0.001
Additional LNs positive	0.9 ± 2.7	0.2 ± 0.9	1.6 ± 3.7	0.6 ± 1.9	2.6 ± 4.2	< 0.001
Total LNs removed	7.8 ± 7.0	3.8 ± 3.5	11.0 ± 7.2	10.5 ± 7.1	13.2 ± 7.4	< 0.001
Total LNs positive	1.5 ± 3.0	0.2 ± 0.9	2.6 ± 3.7	1.6 ± 1.9	4.6 ± 4.2	< 0.001
*Results of ALND*						< 0.001
No additional positive nodes	8268 (72.6)	4716 (93.1)	1629 (59.6)	1372 (73.1)	551 (43.6)	
Additional positive nodes	2675 (24.4)	351 (6.9)	1104 (40.4)	506 (26.9)	714 (56.4)	
*Pathologic nodal stage (overall)*						< 0.001
pN0	78,484 (76.3)	78,038 (98.7)	177 (1.7)	231 (2.3)	38 (1.2)	
pN1	22,691 (22.1)	627 (0.8)	9863 (93.5)	9699 (96.3)	2502 (81.3)	
pN2	867 (0.8)	44 (0.1)	339 (3.2)	97 (1.0)	387 (12.6)	
pN3	336 (0.3)	11 (0.01)	152 (1.4)	26 (0.3)	147 (4.8)	
pNX	85 (0.1)	84 (0.1)	*	*	*	
Unknown	339 (0.3)	302 (0.4)	*	*	*	
*Pathologic nodal stage (SLNB alone)*						< 0.001
pN0	73,754 (80.3)	73367 (99.1)	160 (2.1)	198 (2.4)	29 (1.6)	
pN1	17,664 (19.2)	312 (0.4)	7624 (97.6)	7973 (97.4)	1755 (96.9)	
pN2/3	59 (0.1)	*	19 (0.3)	*	27 (1.5)	
pNX	81 (0.1)	*	*	*	*	
Unknown	293 (0.3)	272 (0.4)	*	10 (0.1)	*	
*Pathologic nodal stage (SLNB then ALND)*						< 0.001
pN0	4730 (43.2)	4671 (92.1)	17 (0.6)	33 (1.8)	9 (0.7)	
pN1	5027 (45.9)	315 (6.2)	2239 (81.9)	1726 (91.9)	747 (59.0)	
pN2	827 (7.6)	41 (0.8)	327 (12.0)	91 (4.9)	368 (29.0)	
pN3	317 (2.9)	10 (0.2)	145 (5.3)	23 (1.2)	139 (11.0)	
pNX	*	*	*	*	*	
Unknown	*	*	*	*	*	

Data are expressed as *n* (%) unless otherwise specified

*NH* non-Hispanic, *HR* hormone receptor, *HER2* human epidermal growth factor receptor 2, *TNBC* triple negative breast cancer

^*^Results suppressed: NCDB does not permit aggregate results for cell sizes < 10

**Fig. 1:**
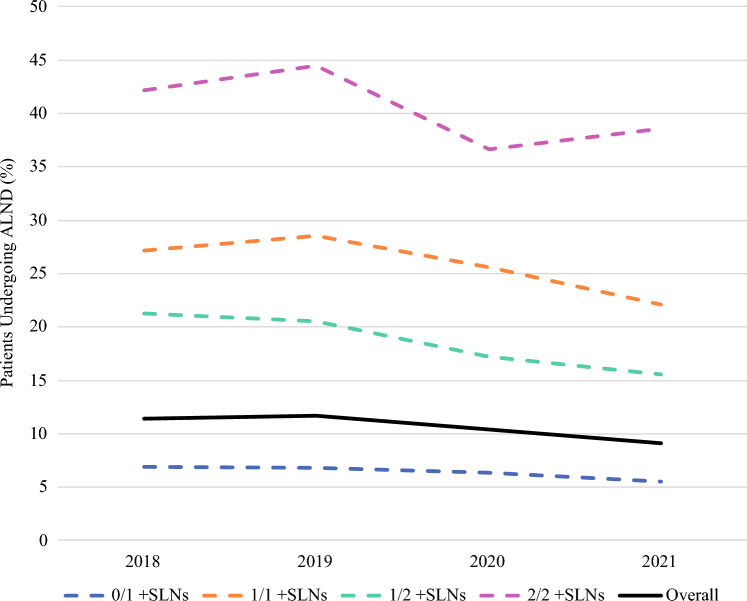
Rates of ALND over time; *ALND* axillary lymph node dissection, *SLNs* sentinel lymph nodes

Overall, 11% of patients underwent a cALND either in the same operation or during a subsequent procedure. When examined over time, the rate of cALND decreased slightly over time (Fig. 1). As expected, cALND was most commonly performed in patients with 2/2 +SLNs (41% versus 0/1: 6%, 1/1: 26%, 1/2: 19%, *p* < 0.001). Among patients in whom cALND was performed, additional +LNs were found in 24% of patients. When examined by SLN group, additional +LNs were found in more than half of patients with 2/2 +SLNs (56%, 714/1267), in 40% (1104/2735) of patients with 1/1 +SLN, and in 27% (506/1878) of patients with 1/2 +SLNs. As expected, it was rare to find any +LNs when cALND was performed in patients with 0/1 +SLN (7%, 351/5071).

Overall, a mean of 6.6 ± 6.8 additional LNs were removed when cALND was performed with 0.9 ± 2.7 additional +LNs found. Interestingly, patients with 0/1 +SLNs had only a mean of 2.8 additional LNs removed at the time of cALND, which was lower than the other SLN groups (1/1: 10.0, 1/2: 8.5, 2/2: 11.2, *p* < 0.001). In line with other findings, the mean number of additional positive nodes found on cALND was highest among patients with 2/2 +SLNs (2.6 versus 0/1: 0.2, 1/1: 1.6, 1/2: 0.6, *p* < 0.001). Overall, 24% of patients upstaged from cN0 to ≥ pN1, something that was more common for patients who had cALND versus SLNB alone (57% versus 20%). Only 1% of patients were ultimately staged as pN2–3. Within the 2/2 +SLN group, 0.4% of patients who had SLNB alone had pN2–3 disease compared with 40% of patients who had cALND. This upstage in the SLNB alone group can be explained either by data entry error or a small proportion of patients with evidence of nodal involvement in basins outside the axilla on subsequent imaging.

### Factors Associated with ALND

On univariate analysis, almost all clinicodemographic and pathologic features had some association with receipt of cALND (Table 3). Subsequent multivariable analysis adjusting for pertinent features found that the strongest independent predictor of cALND was SLN status. Specifically, having 2/2 +SLNs was the strongest predictor (OR 6.8; 95% CI 6.1–7.5), followed by 1/1 +SLNs (OR 3.8; 95% CI 3.5–4.1) and 1/2 +SLNs (OR 2.6; 95% CI 2.4–2.8) when compared with patients with 0/1 +SLNs. Other predictors of ALND were Black race (OR 1.2; 95% CI 1.1–1.3) and Hispanic ethnicity (OR 1.3; 95% CI 1.2–1.4) when compared with non-Hispanic white patients. Additional predictors of ALND included the highest quartile of educational attainment, treatment in a community cancer center, more advanced tumor stage, HER2+ and TNBC subtypes, presence of lymphovascular invasion, and mastectomy as the surgical approach (all *p* < 0.05). Age and tumor histology (ductal versus lobular) were not independent predictors of cALND.

**Table 3:** Univariate and multivariable analysis of factors predictive of ALND receipt

	Univariate			Multivariable		
	OR	95% CI	*p*	OR	95% CI	*p*
Age at diagnosis (years)	0.98	0.98–0.98	< 0.001	1.00	1.00 - 1.00	0.91
*Race/ethnicity*						
NH white (ref.)	–	–	–	–	–	–
NH Black	1.2	1.12–1.28	< 0.001	1.18	1.08–1.30	< 0.001
Hispanic	1.46	1.36–1.58	< 0.001	1.28	1.15–1.41	< 0.001
Asian	1.13	1.03–1.24	0.001	0.99	0.88–1.11	0.84
*Charlson–Deyo score*						
0 (ref.)	–	–	–	–	–	–
1	1.01	0.95–1.07	0.75	0.99	0.91–1.07	0.71
2	0.90	0.81–1.01	0.08	0.88	0.76–1.03	0.11
≥ 3	0.95	0.83–1.08	0.43	0.96	0.81–1.15	0.68
*Insurance status*						
Not insured (ref.)	–	–	–	–	–	–
Private insurance	0.75	0.63–0.90	0.002	0.91	0.72–1.16	0.45
Medicaid	0.94	0.78–1.14	0.54	0.92	0.72–1.19	0.55
Medicare	0.60	0.50–0.72	< 0.001	0.91	0.71–1.16	0.44
Other government	0.75	0.58–0.96	0.02	0.94	0.68–1.32	0.74
*No high school degree*						
≥ 15.3% (ref.)	–	–	–	–	–	–
9.1–15.2%	0.86	0.80–0.92	< 0.001	0.89	0.82–0.96	0.005
5.0–9.0%	0.81	0.76–0.87	< 0.001	0.86	0.79–0.94	< 0.001
< 5.0%	0.73	0.68–0.78	< 0.001	0.79	0.72–0.87	< 0.001
*Median income*						
< $46,277 (ref.)	–	–	–	–	–	–
$46,227–57,856	0.95	0.88–1.03	0.21	1.03	0.94–1.13	0.55
$57,857–74,062	0.94	0.87–1.01	0.09	1.10	1.00–1.21	0.06
≥ $74,063	0.86	0.80–0.92	< 0.001	1.05	0.95–1.16	0.31
*Institution type*						
Community cancer center (ref.)	–	–	–	–	–	–
Comprehensive community cancer program	0.76	0.71–0.82	< 0.001	0.72	0.66–0.79	< 0.001
Academic/research program	0.73	0.68–0.79	< 0.001	0.71	0.64–0.78	< 0.001
Integrated network cancer program	0.70	0.65–0.76	< 0.001	0.66	0.59–0.73	< 0.001
*Clinical tumor stage*						
cT1 (ref.)	–	–	–	–	–	–
cT2	2.18	2.08–2.28	< 0.001	1.17	1.10–1.25	< 0.001
cT3	5.09	4.56–5.69	< 0.001	1.50	1.29–1.74	< 0.001
*Receptor subtype*						
HR+/HER2− (ref.)	–	–	–	–	–	–
HER2+	1.13	1.03–1.25	0.001	1.08	0.95–1.23	0.22
TNBC	1.07	0.99–1.16	0.11	1.16	1.03–1.30	0.01
*Tumor histology*						
Ductal (ref.)	–	–	–	–	–	–
Lobular	1.39	1.31–1.46	< 0.001	1.04	0.96–1.12	0.34
Mixed/other	1.10	0.86–1.40	0.45	0.85	0.60–1.21	0.37
*Tumor grade*						
Low (ref.)	–	–	–	–	–	–
Intermediate	1.57	1.49–1.65	< 0.001	1.09	1.02–1.17	0.01
High	1.91	1.80–2.03	< 0.001	1.20	1.10–1.31	< 0.001
*Lymphovascular invasion*						
Absent (ref.)	–	–	–	–	–	–
Present	2.81	2.68 - 2.94	< 0.001	1.23	1.15 - 1.31	< 0.001
*Breast surgery*						
Partial mastectomy (ref.)	–	–	–	–	–	–
Mastectomy	4.47	4.29–4.65	< 0.001	3.49	3.30–3.68	< 0.001
*SLN status*						
0/1 (ref)	–	–	–	–	–	–
1/1	5.11	4.85–5.38	< 0.001	3.80	3.54–4.08	< 0.001
1/2	3.35	3.16–3.55	< 0.001	2.56	2.37–2.77	< 0.001
2/2	10.21	9.45–11.03	< 0.001	6.76	6.09–7.50	< 0.001

*ALND* axillary lymph node dissection, *NH* non-Hispanic, *HR* hormone receptor, *HER2* human epidermal growth factor receptor 2, *TNBC* triple negative breast cancer

An additional regression model was created to assess factors predictive of finding additional +LNs among patients who underwent cALND. Univariate analysis showed that all tumor and treatment factors, younger age, and institution type were associated with additional +LNs (Table 4). On multivariable analysis, the strongest predictor of having additional +LNs nodes was again SLN status. Odds of finding additional +LNs were ten times higher among patients with 2/2 +SLNs than those with 0/1+ SLNs, six times higher in those with 1/1 +SLNs, and 3.5 times higher in the 1/2 +SLN group. Other independent predictors included presence of lymphovascular invasion (OR 2.2; 95% CI 1.9–2.5), cT2 (OR 1.4; 95% CI 1.3–1.6) and cT3 (OR 1.9; 95% CI 1.4–2.5) stage, mastectomy as the surgical approach (OR 1.6; 95% CI 1.4–1.8), lobular histology (OR 1.4; 95% CI 1.2–1.7), and intermediate (OR 1.3; 95% CI 1.1–1.5) and high (OR 1.3; 95% CI 1.1–1.7) tumor grade.

**Table 4:** Univariate and multivariable analysis of factors associated with finding additional positive nodes among those undergoing ALND

	Univariate			Multivariable		
	OR	95% CI	*p*	OR	95% CI	*p*
Age at diagnosis (years)	0.99	0.99–0.99	< 0.001	1.00	0.99–1.01	0.88
*Race/ethnicity*						
NH white (ref.)	–	–	–	–	–	–
NH Black	0.94	0.81–1.09	0.41	1.09	0.88–1.36	0.42
Hispanic	0.93	0.79–1.10	0.39	0.79	0.62–1.01	0.06
Asian	0.89	0.73–1.09	0.25	0.71	0.54–0.94	0.02
*Charlson–Deyo score*						
0 (ref)	–	–	–	–	–	–
1	1.00	0.88–1.14	0.99	0.96	0.79–1.16	0.69
2	1.06	0.83–1.36	0.63	1.20	0.85–1.70	0.30
≥ 3	0.89	0.65–1.21	0.44	0.98	0.64–1.49	0.91
*Insurance status*						
Not insured (ref.)	–	–	–	–	–	–
Private insurance	1.06	0.72–1.56	0.75	1.38	0.80–2.37	0.25
Medicaid	1.24	0.82–1.87	0.30	1.77	0.99–3.16	0.05
Medicare	0.93	0.63–1.37	0.70	1.44	0.83–2.52	0.20
Other government	1.02	0.59–1.76	0.96	1.00	0.46–2.17	0.99
*No high school degree*						
≥ 15.3% (ref)	–	–	–	–	–	–
9.1–15.2%	1.01	0.87–1.16	0.92	0.98	0.81–1.19	0.84
5.0–9.0%	0.95	0.82–1.09	0.44	0.98	0.80–1.21	0.86
< 5.0%	0.87	0.75–1.01	0.06	0.94	0.74–1.19	0.61
*Median income*						
< $46,277 (ref.)	–	–	–	–	–	–
$46,227–57,856	0.95	0.80–1.12	0.51	0.96	0.77–1.20	0.74
$57,857–74,062	0.92	0.78–1.07	0.28	0.93	0.74–1.16	0.50
≥ $74,063	0.86	0.74–0.99	0.04	0.84	0.66–1.06	0.13
*Institution type*						
Community cancer center (ref.)	–	–	–	–	–	–
Comprehensive community cancer program	1.21	1.03–1.43	0.02	0.96	0.76–1.21	0.73
Academic/research program	1.29	1.08–1.53	0.00	0.96	0.75–1.22	0.74
Integrated network cancer program	1.24	1.04–1.49	0.02	0.93	0.72–1.20	0.57
*Clinical tumor stage*						
cT1 (ref.)	–	–	–	–	–	–
cT2	2.46	2.24–2.70	< 0.001	1.43	1.25–1.64	< 0.001
cT3	5.06	4.19–6.10	< 0.001	1.91	1.41–2.47	< 0.001
*Receptor subtype*						
HR+/HER2− (ref.)	–	–	–	–	–	–
HER2+	0.89	0.72–1.09	0.26	1.02	0.76–1.38	0.88
TNBC	0.54	0.44–0.67	< 0.001	0.83	0.61–1.11	0.21
*Tumor histology*						
Ductal (ref.)						-
Lobular	1.78	1.59–1.98	< 0.001	1.40	1.19–1.65	< 0.001
Mixed/other	0.66	0.36–1.23	0.19	0.81	0.33–2.02	0.65
*Tumor grade*						
Low (ref.)	–	–	–	–	–	–
Intermediate	2.02	1.78–2.30	< 0.001	1.26	1.05–1.51	0.02
High	2.08	1.80–2.41	< 0.001	1.34	1.08–1.67	0.01
*Lymphovascular invasion*						
Absent (ref)	–	–	–	–	–	–
Present	4.36	3.95 - 4.81	< 0.001	2.17	1.90 - 2.47	< 0.001
*Breast surgery*						
Partial mastectomy (ref.)	–	–	–	–	–	–
Mastectomy	2.86	2.60–3.15	< 0.001	1.58	1.37–1.81	< 0.001
*SLN status*	0.00	–		0.00	–	
0/1 (ref)	–	–	–	–	–	–
1/1	9.11	7.97–10.40	< 0.001	6.01	5.02–7.20	< 0.001
1/2	4.96	4.27–5.75	< 0.001	3.49	2.86–4.25	< 0.001
2/2	17.41	14.91–20.34	< 0.001	10.05	8.16–12.38	< 0.001

*ALND* axillary lymph node dissection, *NH* non-Hispanic, *HR* hormone receptor, *HER2* human epidermal growth factor receptor 2, *TNBC* triple negative breast cancer

### Receptor Subgroup Analysis

After stratifying the cohort by receptor subtype, the use of adjuvant therapies was compared between pN+ patients undergoing SLNB alone and cALND in each SLN group. Among patients with HER2+ or TNBC, cALND was not associated with differences in the rates of adjuvant chemotherapy or radiation therapy for nearly all groups (Supplementary Tables 1, 2). The only observed difference was a lower rate of adjuvant radiation among patients who had cALND in both patient with HER2+ and TNBC (*p* = 0.02 and *p* = 0.003, respectively), though these differences did not persist after stratifying by breast surgical approach, likely reflecting the higher rate of mastectomy in the cALND group. The major differences in adjuvant therapy were seen in pN+ HR+/HER2− patients (Supplementary Table 3). Overall, adjuvant chemotherapy was most commonly given to patients with 2/2 +SLNs who underwent cALND (65%) and least in those with 1/1+ SLNs who had SLNB alone (28%), with significant differences noted when comparing SLN groups and axillary surgical approach (both *p* < 0.001).

Since current clinical practice supports using the 21-gene recurrence score (RS) to guide systemic therapy only among pN+ patients who are post-menopausal, we created a cohort of the 16,288 HR+/HER2− patients > 50 years old for further analysis (Table 5). Within this group, RS testing was performed in 55% of patients and was less common among patients who had cALND and 2/2 +SLNs (both *p* < 0.001). The mean RS of the cohort was 16, with > 80% of patients in each group having RS ≤ 25. There was no difference in mean RS seen when comparing SLN group (*p* = 0.55) or by ALND receipt (*p* = 0.43). Despite this lack of difference in RS in node-positive HR+/HER2− patients > 50, those who underwent cALND had a higher rate of adjuvant chemotherapy in all SLN groups (1/1: 40% versus 23%; 1/2: 33% versus 22%; 2/2: 60% versus 35%; all *p* < 0.0001). When the cohort was stratified by pathologic nodal stage, this higher rate of adjuvant chemotherapy for cALND persisted in patients with pN1 disease (*p* < 0.001), but not those with pN2 disease (*p* = 0.69, data not shown). Similar to patients with HER2+ and TNBC, those who had cALND were less likely to undergo adjuvant radiotherapy (*p* < 0.001).

**Table 5:** Adjuvant therapies among pN+ patients > 50 with HR+/HER2− breast cancer

	Sentinel lymph node status						*p*^1^	*p*^2^
	1/1		1/2		2/2			
	SLNB only	cALND	SLNB only	cALND	SLNB only	cALND		
*n*	5659	1763	5648	1114	1288	816		
*Recurrence score*								
Not performed	2297 (40.6)	1017 (57.7)	2217 (39.3)	532 (47.8)	641 (49.8)	579 (71.0)	< 0.001	< 0.001
Performed	3362 (59.4)	746 (42.3)	3431 (60.8)	582 (52.2)	647 (50.2)	237 (29.0)		
Mean ± SD	16.2 ± 9.3	16.2 ± 9.2	16.0 ± 9.0	16.6 ± 10.1	15.8 ± 8.4	15.9 ± 9.1	0.55	0.43
Score ≤ 25	2816 (83.76)	633 (84.85)	2908 (84.76)	472 (81.10)	567 (87.64)	200 (84.39)	0.11	0.09
Score > 25	436 (12.97)	90 (12.06)	401 (11.69)	94 (16.15)	61 (9.43)	30 (12.66)		
*Chemotherapy*							< 0.001	< 0.001
No/unknown	4567 (80.7)	1050 (59.6)	4416 (78.2)	742 (66.6)	841 (65.3)	326 (40.0)		
Yes	1272 (22.5)	713 (40.4)	1232 (21.8)	372 (33.4)	447 (34.7)	490 (60.1)		
*Endocrine therapy*							0.12	< 0.001
No	447 (7.9)	182 (10.3)	423 (7.5)	96 (8.6)	108 (8.4)	76 (9.3)		
Yes	5145 (90.9)	1548 (87.8)	5150 (91.2)	992 (89.1)	1152 (89.4)	721 (88.4)		
Unknown	67 (1.2)	33 (1.9)	75 (1.3)	26 (2.3)	28 (2.2)	19 (2.3)		
*Radiation: all patients*							< 0.001	< 0.001
No	835 (14.8)	600 (34.0)	902 (16.0)	386 (34.7)	153 (11.9)	163 (20.0)		
Yes	4756 (84.0)	1137 (64.5)	4671 (82.7)	707 (63.5)	1106 (85.9)	638 (78.2)		
Unknown	68 (1.2)	26 (1.5)	75 (1.3)	21 (1.9)	29 (2.3)	15 (1.8)		
*Radiation: mastectomy patients*							< 0.001	0.23
No	510 (42.4)	533 (45.9)	587 (44.1)	347 (49.7)	80 (22.2)	137 (25.6)		
Yes	671 (55.8)	614 (52.8)	721 (54.1)	337 (48.3)	267 (74.2)	388 (72.4)		
Unknown	21 (1.8)	15 (1.3)	24 (1.8)	14 (2.0)	13 (3.6)	11 (2.1)		
*Radiation: lumpectomy patients*							< 0.001	< 0.001
No	325 (7.3)	67 (11.2)	315 (7.3)	39 (9.4)	73 (7.9)	26 (9.3)		
Yes: breast only	1730 (38.8)	185 (30.8)	1725 (40.0)	149 (35.8)	241 (26.0)	49 (17.5)		
Yes: breast and LNs	2146 (48.2)	298 (49.6)	2018 (46.8)	193 (46.4)	543 (58.5)	180 (64.3)		
Yes: other	181 (4.1)	35 (5.8)	182 (4.2)	*	49 (5.3)	*		
Unknown	75 (1.7)	16 (2.7)	76 (1.8)	*	22 (2.4)	*		

Data are expressed as *n* (%) unless otherwise specified.

*pN* pathological nodal stage, *HR* hormone receptor, *HER2* human epidermal growth factor receptor 2, *TNBC* triple negative breast cancer, *SLNB* sentinel lymph node biopsy, *cALND* completion axillary lymph node dissection, *LN* lymph node,* p*^*1*^ comparison between LN groups,* p*^*2*^ comparison between no ALND versus ALND

^*^Results suppressed: NCDB does not permit aggregate results for cell sizes < 10

### Overall Survival

**Fig. 2:**
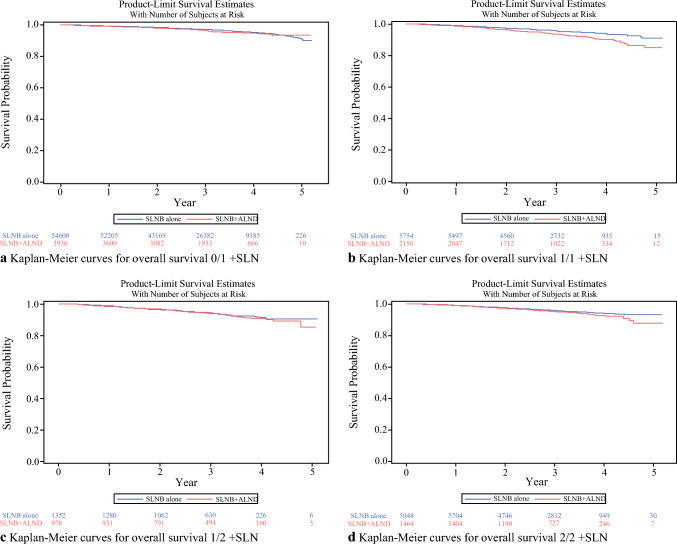
Kaplan–Meier curves for overall survival comparing SLNB alone versus SLNB+ALND for each SLN group; **A** Kaplan–Meier curves for overall survival 0/1 +SLN, **B** Kaplan–Meier curves for overall survival 1/1 +SLN, **C** Kaplan–Meier curves for overall survival 1/2 +SLN, **D** Kaplan–Meier curves for overall survival 2/2 +SLN; *SLNB* sentinel lymphadenectomy, *ALND* axillary lymph node dissection, *SLN* sentinel lymph node

We compared unadjusted OS between patients who did and did not undergo cALND for each SLN group. With a median follow up of 35.4 months, 5-year OS was ≥ 90% in every group, and cALND was not associated with improved OS in any SLN group (Fig. 2). The only difference we observed in unadjusted OS was in the 1/1 +SLN group, where patients undergoing cALND had a worse OS (*p* < 0.001).

## Discussion

In this analysis of a large national database, we found that ALND is being performed at higher-than-expected rates in patients found to have ≤ 2 SLNs retrieved, where all are positive. This behavior flouts randomized evidence demonstrating the safety of ALND omission in this situation. This therapeutic excess may extend to overtreatment in the adjuvant setting for some patients without providing any survival benefit.

To date, this is the first study that evaluates patients who fall into a poorly defined area of axillary management: those in whom ≤ 3 SLNs are retrieved and all are positive. Due to concerns for additional axillary disease in these patients, surgeons (and others) often question whether ALND should be performed for local control and/or to change adjuvant treatment recommendations if more disease is found. This is likely the reason we found that SLN status was the strongest predictor of cALND, surpassing any other clinicopathologic factor. The extent of the ALNDs being performed does need to be questioned given the low number of additional LNs, as most cALND should include > 10 additional LNs, especially in the non-neoadjuvant patient. Perhaps the extent of ALND performed was influenced by the surgeon’s expectation of finding additional disease. This contradicts data from Z0011 and AMAROS that demonstrated the safety of ALND omission despite additional axillary disease found when ALND was performed in 27% and 33% of patients, respectively. ^5,6^ Overall, our analysis demonstrated a similar proportion of additional axillary disease (24%) in patients who underwent cALND. Although many would consider the patients in our cohort to be at higher risk for harboring additional axillary disease than those enrolled in the randomized trials (many of whom likely had negative SLNs retrieved), these comparable findings suggest that applying the paradigms of cALND omission is reasonable. We did find that 40% of patients with 1/1 +SLN and more than half of patients in the 2/2 +SLN group had additional axillary disease, which is similar to the findings in the SENOMAC trial, where 31% of patients had additional axillary disease in patients with one +SLN and 51% of patients with two +SLNs.^8^ This does give some credence to the concern of surgeons about leaving behind clinically significant disease in these patients. Therefore, cALND should be considered on a case-by-case basis in these patients after a thorough multidisciplinary review of risks and benefits.

Any decision to perform a cALND for the perceived benefit in improved local and distant control needs to be weighed against the risks that come with the operation. The increased morbidity of ALND, specifically the risk of lymphedema, but also of seroma formation, paresthesias, infection, and decreased shoulder mobility, should not be taken lightly. Lymphedema may be associated with decreased OS in patients with breast cancer.^14^ The incidence of arm lymphedema in women with breast cancer is approximately 16%, but is estimated to be four times higher in women who undergo ALND versus those who undergo SLNB alone.^15^ This has been a driving force behind continued studies on de-escalation of axillary treatment over the past 30 years. Additionally, Black race and Hispanic ethnicity have been identified as independent risk factors in the development of lymphedema.^16^ In our study, both Black race and Hispanic ethnicity were independent predictors of ALND, but not of finding additional positive nodes. Thus, these already-vulnerable patient populations are at a higher risk for surgical morbidity from ALND without deriving additional benefit, and surgeon education to reduce avoidable axillary overtreatment is crucial.

Multidisciplinary teams may also advocate for cALND due to the perceived benefit that finding additional positive nodes would result in changing adjuvant therapy recommendations on the basis of the findings. While the surgical approach may change the adjuvant radiation therapy field planning and volume of nodal disease for some radiation oncologists, it is unlikely that pN1 versus ≥ pN2 disease changes whether adjuvant radiation is considered.^17^ This was confirmed in our analysis, as we found that ALND did not affect rates of adjuvant radiation in this study.

Similarly, recommendations for adjuvant chemotherapy administration are unlikely to differ between pN1 and ≥ pN2 disease in patients with HER2+ and TNBC, and in pre-menopausal patients with HR+/HER2− disease. Our results confirm adherence to these treatment paradigms, with similar rates of adjuvant chemotherapy in these groups whether cALND was performed or not. While the receipt of adjuvant CDK4/6 inhibition is often based on the total number of positive nodes in patients with HR+/HER2− disease, we have previously demonstrated that cALND should not be routinely used to determine candidacy for this adjunctive therapy.^18,19^ One subgroup where pN upstaging could affect adjuvant chemotherapy decision-making is in post-menopausal patients with HR+/HER2− disease where ≥ pN2 patients are recommended adjuvant chemotherapy but pN1 patients are only recommended adjuvant chemotherapy if they are found to be at high risk based on genomic testing.^17^ We studied this specific cohort and found that despite similar recurrence scores, patients in the cALND group had higher rates of adjuvant chemotherapy use even among patients who remained stage pN1. This suggests that performance of ALND is associated with adjuvant chemotherapy overtreatment in this population.

While this study and a recent study examining predictors of pN2 disease found that the strongest predictor of finding additional positive nodes with cALND was SLN status, there were several additional predictors of finding additional positive nodes that can be used as considerations when estimating its benefit in specific situations.^20^ These included lymphovascular invasion, cT2 and cT3 stage, mastectomy as the surgical approach, lobular histology, and intermediate and high tumor grade. These factors are similar to those used to create the additional non-SLN metastases nomogram from Memorial Sloan Kettering Cancer Center.^21,22^ Katz et al. found that increasing primary tumor size, histologic grade, number of +SLNs, and size of the largest SLN metastasis were the most predictive of upstaging to pN2 or greater, while an increasing number of uninvolved SLNs was protective against upstaging.^23^ Given the high likelihood of finding additional axillary disease and pN upstaging when 2/2 +SLNs are retrieved, it is suggested that in HR+/HER2− patients > 50 years old with RS ≤ 25, patients with 2/2 +SLNs be discussed in a multidisciplinary tumor board regarding the possible benefits of undergoing cALND. Unfortunately, due to the small number of patients, attempts at analyzing this cohort for changes in adjuvant treatments were underpowered.

Local control with surgery and optimized adjuvant therapies are all employed toward the goal of improving disease-free survival and OS but need to be right sized for each patient and used only when there will be positive impact. From the evaluation in NSABP B-04 of radical mastectomy versus total mastectomy to more modern trials of axillary de-escalation, such as SINODAR-ONE and SENOMAC, there has been a consistent theme that no randomized control trial has ever shown a survival benefit from ALND. In line with these prospective studies, we found no evidence that cALND improved survival in this analysis and therefore should not be pursued in patients for whom this is the only goal of additional surgery.

This study has several strengths and limitations. Its major strength is the use of a large, national database that permitted us to investigate current practice patterns in this patient population. Additionally, data regarding the number of sentinel and non-sentinel nodes that were removed and positive allowed us to analyze the incremental benefit of cALND when performed. The study is limited by its retrospective nature and lack of patient-specific decision-making data points. Specifically, ECE is missing and this may factor into some providers’ decision-making as it was part of the exclusion criteria in Z0011; although ECE was not excluded in the SINODAR-ONE and SENOMAC trials.^7,8^ In addition, the NCDB lacks cancer-specific outcomes such as disease-free survival along with rates of local, regional, and distance recurrence, which would be better endpoints when evaluating the efficacy of treatments. Additionally, we attempted to evaluate and compare OS in HR+/HER2− patients > 50 with pN1 versus ≥ pN2 disease who had a SLNB alone versus ALND, but due to small numbers in this cohort this analysis was underpowered. Therefore, this is something that should be further investigated. Lastly, due to SLNB-specific information not reported in NCDB until 2018, this study lacks long-term follow-up, as the median was only 35.4 months. Future work with longer follow-up and breast-cancer-specific outcomes is needed.

## Conclusions

The criteria for ALND omission defined by both ACOSOG Z0011 and AMAROS can and should be followed for patients in whom fewer than 3 SLNs are retrieved. No further axillary surgery should be pursued in patients with 1 +SLN, but a detailed multidisciplinary discussion weighing the surgical morbidity and the potential benefits of local control and adjuvant therapy should be undertaken for patients with 2/2 +SLNs.

## Supplementary Information

Below is the link to the electronic supplementary material.

Supplementary file1 (DOCX 130 kb)
