# Supplementary material for: Management and Outcomes in Patients with Breast Cancer with 1-of-1 and 2-of-2 Positive Sentinel Nodes
Source: Ann Surg Oncol. 2025 Aug 23;32(13):9843–56. doi: 10.1245/s10434-025-18097-9 (PMC12589349; doi:10.1245/s10434-025-18097-9)
Supplement: Supplementary file 1 — Supplementary file1 (DOCX 130 kb) [file 10434_2025_18097_MOESM1_ESM.docx]

**Supplementary Tables and Figures**

**Supplementary Figure 1: Strobe Diagram**


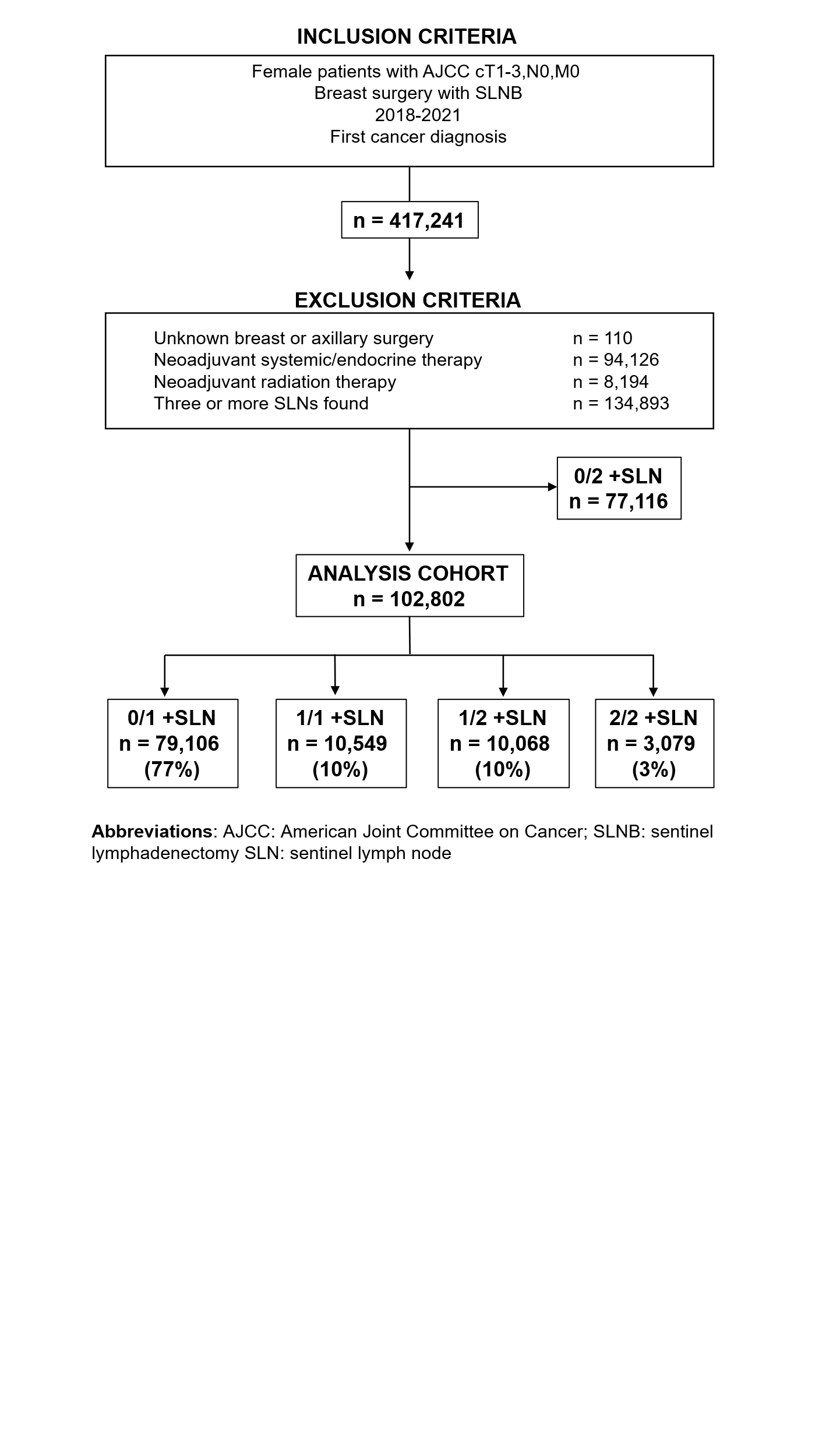


**Supplementary Table 1: Adjuvant therapies among pN+ patients with triple negative breast cancer**

|  | **1/1** | | | **1/2** | | | **2/2** | | | **p1** | **p2** |
| --- | --- | --- | --- | --- | --- | --- | --- | --- | --- | --- | --- |
|  | **No ALND** | **ALND** | **p** | **No ALND** | **ALND** | **p** | **No ALND** | **ALND** | **p** |  |  |
| **n** | **268** | **117** |  | **306** | **86** |  | **77** | **42** |  |  |  |
| **Chemotherapy** |  |  | **0.61** |  |  | **0.37** |  |  | **0.65** | **0.48** | **0.67** |
| **No/Unknown** | **51 (19.0)** | **20 (17.1)** |  | **56 (18.3)** | **19 (22.1)** |  | **16 (20.8)** | **11 (26.2)** |  |  |  |
| **Yes** | **217 (81.0)** | **97 (82.9)** |  | **250 (81.7)** | **66 (76.7)** |  | **61 (79.2)** | **31 (73.8)** |  |  |  |
| **Radiation - all patients** |  |  | **0.001** |  |  | **0.06** |  |  | **0.51** | **0.21** | **0.003** |
| **No/Unknown** | **61 (22.7)** | **52 (37.6)** |  | **89 (29.1)** | **34 (39.6)** |  | **21 (27.1)** | **13 (31.0)** |  |  |  |
| **Yes** | **207 (77.2)** | **73 (62.4)** |  | **217 (70.9)** | **52 (60.5)** |  | **56 (72.7)** | **29 (69.1)** |  |  |  |
| **Radiation - mastectomy patients** |  |  | **0.40** |  |  | **0.27** |  |  | **0.93** | **0.06** | **0.13** |
| **No/Unknown** | **28 (43.1)** | **29 (36.7)** |  | **47 (59.5)** | **29 (49.2)** |  | **10 (38.5)** | **12 (42.9)** |  |  |  |
| **Yes** | **33 (50.8)** | **46 (58.2)** |  | **32 (40.5)** | **29 (49.2)** |  | **14 (53.9)** | **16 (57.1)** |  |  |  |
| **Radiation - lumpectomy patients** |  |  | **0.54** |  |  | **0.67** |  |  | **0.83** | **0.002** | **0.66** |
| **No** | **27 (13.3)** | ***** |  | **38 (16.7)** | ***** |  | ***** | ***** |  |  |  |
| **Yes - breast only** | **51 (25.1)** | ***** |  | **62 (27.3)** | **8 (29.6)** |  | ***** | ***** |  |  |  |
| **Yes - breast and LNs*** | **102 (50.3)** | **15 (39.5)** |  | **112 (49.3)** | **12 (44.4)** |  | **23 (45.1)** | ***** |  |  |  |
| **Yes - other** | ***** | ***** |  | ***** | ***** |  | **10 (19.6)** | ***** |  |  |  |
| **Unknown** | ***** | ***** |  | ***** | ***** |  | ***** | ***** |  |  |  |

Data are expressed as *n* (%) unless otherwise specified

ALND: axillary lymph node dissection; LNs: lymph nodes

*Results suppressed: NCDB does not permit aggregate results for cell sizes < 10

**Supplementary Table 2: Adjuvant therapies among pN+ patients with HER2+ breast cancer**

|  | **1/1** | | | **1/2** | | | **2/2** | | | **p1** | **p2** |
| --- | --- | --- | --- | --- | --- | --- | --- | --- | --- | --- | --- |
|  | **No ALND** | **ALND** | **p** | **No ALND** | **ALND** | **p** | **No ALND** | **ALND** | **p** |  |  |
| **n** | **249** | **115** |  | **255** | **74** |  | **66** | **47** |  |  |  |
| **Chemotherapy** |  |  | **0.70** |  |  | **0.60** |  |  | **0.04** | **0.76** | **0.43** |
| **No/Unknown** | **104 (41.8)** | **46 (40.0)** |  | **98 (38.5)** | **30 (40.5)** |  | **29 (43.9)** | **12 (25.5)** |  |  |  |
| **Yes** | **145 (58.2)** | **69 (60.0)** |  | **157 (61.6)** | **44 (59.5)** |  | **37 (56.1)** | **35 (74.5)** |  |  |  |
| **Radiation - all patients** |  |  | **0.07** |  |  | **0.16** |  |  | **0.63** | **0.75** | **0.02** |
| **No/Unknown** | **90 (36.1)** | **53 (46.1)** |  | **89 (34.9)** | **31 (41.9)** |  | **21 (31.8)** | **17 (36.2)** |  |  |  |
| **Yes** | **159 (63.9)** | **62 (53.9)** |  | **166 (65.1)** | **43 (58.1)** |  | **45 (68.2)** | **30 (63.8)** |  |  |  |
| **Radiation - mastectomy patients** |  |  | **0.73** |  |  | **0.56** |  |  | **0.68** | **0.2212** | **0.31** |
| **No/Unknown** | **49 (57.0)** | **42 (54.6)** |  | **43 (58.2)** | **27 (51.9)** |  | **10 (45.5)** | **16 (40.0)** |  |  |  |
| **Yes** | **37 (43.0)** | **35 (45.5)** |  | **31 (41.9)** | **25 (48.1)** |  | **12 (54.6)** | **24 (60.0)** |  |  |  |
| **Radiation - lumpectomy patients** |  |  | **0.21** |  |  | **0.70** |  |  | **0.51** | **0.96** | **0.59** |
| **No** | **34 (20.9)** | ***** |  | **39 (21.6)** | ***** |  | **11 (25.0)** | ***** |  |  |  |
| **Yes - breast only** | **39 (23.9)** | ***** |  | **50 (27.6)** | ***** |  | **11 (25.0)** | ***** |  |  |  |
| **Yes - breast and LNs*** | **77 (47.2)** | **13 (34.2)** |  | **74 (40.9)** | **12 (54.6)** |  | **19 (43.2)** | ***** |  |  |  |
| **Yes - other** | ***** | ***** |  | ***** | ***** |  | ***** | ***** |  |  |  |
| **Unknown** | ***** | ***** |  | ***** | ***** |  | ***** | ***** |  |  |  |

Data are expressed as *n* (%) unless otherwise specified

HER2: human epidermal growth factor 2; ALND: axillary lymph node dissection; LNs: lymph nodes

*Results suppressed: NCDB does not permit aggregate results for cell sizes < 10

**Supplementary Table 3: Adjuvant therapies among pN+ patients with HR+/HER2- breast cancer**

|  | **1/1** | | | **1/2** | | | **2/2** | | | **p1** | **p2** |
| --- | --- | --- | --- | --- | --- | --- | --- | --- | --- | --- | --- |
|  | **No ALND** | **ALND** | **p** | **No ALND** | **ALND** | **p** | **No ALND** | **ALND** | **p** |  |  |
| **n** | **7021** | **2443** |  | **7327** | **1655** |  | **1616** | **1147** |  |  |  |
| **Recurrence Score** |  |  |  |  |  |  |  |  |  |  |  |
| **Not performed** | **2888 (41.13)** | **1415 (57.92)** | **<0.001** | **2947 (40.22)** | **829 (50.09)** | **<0.001** | **834 (51.61)** | **809 (70.53)** | **<0.001** | **<0.001** | **<0.001** |
| **Performed** | **4133 (58.87)** | **1028 (42.08)** |  | **4380 (59.78)** | **826 (49.91)** |  | **782 (48.39)** | **338 (29.47)** |  |  |  |
| **Mean ± S.D.** | **16.32 ± 9.08** | **16.73 ± 9.45** | **0.2100** | **16.25 ± 8.94** | **17.10 ± 10.02** | **0.03** | **15.97 ± 8.25** | **16.84 ± 10.00** | **0.17** | **0.86** | **0.01** |
| **Chemotherapy** |  |  | **<0.001** |  |  | **<0.001** |  |  | **<0.001** | **<0.001** | **<0.001** |
| **No/Unknown** | **5085 (72.4)** | **1280 (52.4)** |  | **5235 (71.4)** | **953 (57.6)** |  | **945 (58.5)** | **398 (34.7)** |  |  |  |
| **Yes** | **1936 (27.57)** | **1163 (47.61)** |  | **2092 (28.55)** | **702 (42.42)** |  | **671 (41.52)** | **749 (65.30)** |  |  |  |
| **Endocrine therapy** |  |  | **0.01** |  |  | **0.10** |  |  | **0.82** | **0.01** | **0.001** |
| **No** | **548 (7.81)** | **234 (9.58)** |  | **534 (7.29)** | **139 (8.40)** |  | **148 (9.16)** | **102 (8.89)** |  |  |  |
| **Yes** | **6366 (90.67)** | **2164 (88.58)** |  | **6690 (91.31)** | **1481 (89.49)** |  | **1435 (88.80)** | **1019 (88.84)** |  |  |  |
| **Unknown** | **107 (1.52)** | **45 (1.84)** |  | **103 (1.41)** | **35 (2.11)** |  | **33 (2.04)** | **26 (2.27)** |  |  |  |
| **Radiation - all patients** |  |  | **<0.001** |  |  | **<0.001** |  |  | **<0.001** | **<0.001** | **<0.001** |
| **No** | **1060 (15.10)** | **785 (32.13)** |  | **1179 (16.09)** | **548 (33.11)** |  | **192 (11.88)** | **229 (19.97)** |  |  |  |
| **Yes** | **5869 (83.59)** | **1614 (66.07)** |  | **6046 (82.52)** | **1080 (65.26)** |  | **1390 (86.01)** | **901 (78.55)** |  |  |  |
| **Unknown** | **92 (1.31)** | **44 (1.80)** |  | **102 (1.39)** | **27 (1.63)** |  | **34 (2.10)** | **17 (1.48)** |  |  |  |
| **Radiation - mastectomy patients** |  |  | **0.13** |  |  | **0.05** |  |  | **0.19** | **<0.001** | **0.19** |
| **No** | **687 (38.92)** | **709 (41.49)** |  | **822 (39.48)** | **503 (44.67)** |  | **106 (21.07)** | **201 (24.51)** |  |  |  |
| **Yes** | **1045 (59.21)** | **970 (56.76)** |  | **1220 (58.60)** | **603 (53.55)** |  | **382 (75.94)** | **606 (73.90)** |  |  |  |
| **Unknown** | **33 (1.87)** | **30 (1.76)** |  | **40 (1.92)** | **20 (1.78)** |  | **15 (2.98)** | **13 (1.59)** |  |  |  |
| **Radiation - lumpectomy patients** |  |  | **<0.001** |  |  | **0.07** |  |  | **0.01** | **<0.001** | **<0.001** |
| **No** | **373 (7.10)** | **76 (10.35)** |  | **357 (6.81)** | **45 (8.51)** |  | **86 (7.73)** | **28 (8.56)** |  |  |  |
| **Yes - breast only** | **1992 (37.90)** | **229 (31.20)** |  | **2050 (39.08)** | **178 (33.65)** |  | **282 (25.34)** | **58 (17.74)** |  |  |  |
| **Yes - breast and LNs** | **2561 (48.73)** | **360 (49.05)** |  | **2517 (47.99)** | **266 (50.28)** |  | **660 (59.30)** | **207 (63.30)** |  |  |  |
| **Yes - other** | **232 (4.41)** | **48 (6.54)** |  | **228 (4.35)** | **28 (5.29)** |  | **56 (5.03)** | ***** |  |  |  |
| **Unknown** | **98 (1.86)** | **21 (2.86)** |  | **93 (1.77)** | **12 (2.27)** |  | **29 (2.61)** | ***** |  |  |  |

Data are expressed as *n* (%) unless otherwise specified

HR: hormone receptor; HER2: human epidermal growth factor 2; ALND: axillary lymph node dissection; LNs: lymph nodes

*Results suppressed: NCDB does not permit aggregate results for cell sizes < 10
